# Enhanced FISH Image Classification via CBAM-PPM-Optimized ResNet50 for Precision Cytogenetic Diagnosis

**DOI:** 10.3390/s25226951

**Published:** 2025-11-13

**Authors:** Zhiling Li, Wenjia Li, Yang Zhou, Liu Wang

**Affiliations:** 1 College of Computer Science and Technology, Changchun University, Changchun 130000, China; 2 The Key Laboratory of Intelligent Rehabilitation and Barrier-Free, Disabled Ministry of Education, Changchun University, Changchun 130000, China; 3 Jilin Provincial Key Laboratory of Human Health Status Identification & Function Enhancement, Changchun 130000, China

**Keywords:** fluorescence in situ hybridization (FISH), ResNet50, Convolutional Block Attention Module (CBAM), Pyramid Pooling Module (PPM)

## Abstract

To address the low efficiency and high subjectivity of manual interpretation in fluorescence in situ hybridization (FISH) tissue and cell images, this study proposes an intelligent FISH image classification model based on an improved ResNet50 architecture. By analyzing the characteristics of multi-channel fluorescence signals and the bottlenecks of clinical interpretation, a Convolutional Block Attention Module (CBAM) is introduced to enhance the representation of salient fluorescence features through dual channel–spatial attention mechanisms. A Pyramid Pooling Module (PPM) is integrated to fuse multi-scale contextual information, improving the detection accuracy of small targets such as microdeletions. Furthermore, the shortcut connections in residual blocks are optimized to reduce feature loss. To mitigate the limitation of insufficient annotated samples, transfer learning is employed, combined with a focal loss function to enhance classification performance under class-imbalanced conditions. Experiments conducted on a clinical dataset of 12,000 FISH images demonstrate that the proposed model achieves an overall classification accuracy of 92.4%, representing a 9.9% improvement over the original ResNet50. The recall rate for complex categories (e.g., translocation and fusion) exceeds 90.7%, with an inference time of 22.3 ms per sample, meeting the real-time requirements of clinical diagnosis. These results provide an efficient and practical solution for the automated intelligent interpretation of FISH images, offering significant potential for precision-assisted diagnosis of tumors and genetic disorders.

## 1. Introduction

FISH, as an essential tool in molecular cytogenetics, enables the localization and detection of gene and chromosomal abnormalities through specific hybridization between fluorescently labeled nucleic acid probes and target sequences within cells or tissues [1]. This technique has demonstrated significant advantages in the early diagnosis of tumors, prenatal genetic screening, and the detection of hereditary diseases, making it a critical component of the precision medicine framework [2,3].

Traditional FISH image analysis still relies heavily on manual interpretation, which suffers from low efficiency, high subjectivity, and poor consistency, making it difficult to meet the demands of large-scale clinical screening and rapid diagnosis. Deep learning-based automated image analysis techniques provide a novel solution to overcome these limitations. Residual networks (ResNet) effectively alleviate the gradient vanishing problem through the introduction of residual connections, enabling the extraction of multi-level semantic features from complex medical images [4]. This provides strong feature representation capabilities for the automatic recognition and classification of FISH images. Compared with traditional texture analysis and morphological methods, ResNet offers higher classification accuracy and robustness, while supporting end-to-end learning and inference [5].

In recent years, significant progress has been made by researchers in FISH image analysis and the optimization of ResNet architectures, such as: Xue T. [6] utilized clinical FISH (HER2) images to build a deep learning model for the automatic classification of HER2 amplification status, demonstrating both the clinical feasibility and limitations of AI-assisted FISH interpretation. Jian Z. [7] adopted an improved U-Net/Unet++ variant integrated with attention mechanisms, focusing on FISH cell contour segmentation and the separation of adherent cells, thus supporting the “attention + segmentation” research direction. Jian Z. [8] proposed an enhanced small-object detection method based on the YOLO series for detecting fine FISH signals, integrating modules such as channel attention and ECA, and provided public datasets and implementation details—valuable for small-object detection and attention-based enhancement strategies. Xu X. [9] developed a lightweight multi-channel (4-color) FISH detection and recognition framework (FISH-Net), covering signal normalization, heatmap refinement, and cross-center validation, offering a useful reference for multi-channel fusion and lightweight deployment. Yan C. [10] demonstrated the potential of combining phenotypic information with weakly supervised or multi-instance learning for pathology and HER2 interpretation, which provides methodological insights for weakly labeled or weakly supervised FISH analysis. Xu W. [11] proposed a general FISH spot detection and enhancement framework (based on U-Net) and built a large-scale FISH spot dataset, emphasizing generalization and 3D processing capabilities across multi-source and multi-modal FISH data. Al T.A.E. [12] analyzed FISH images, and SNP chip analysis of detected abnormalities reflected real cellular variations.

Recent studies have increasingly focused on introducing attention mechanisms and multimodal fusion techniques—such as using CBAMs to enhance detail recognition of fluorescent signals or integrating FISH images with histopathological data to improve classification performance. For example, Godbin A.B. [13] integrated CBAM with a lightweight backbone in chest X-ray and pulmonary disease detection tasks, reporting significant improvements in classification performance and interpretability, supporting the conclusion that “CBAM enhances lesion detail recognition and interpretability in medical images.” Similarly, Pang B. [14] embedded CBAM into a lightweight U-Net for medical image segmentation and validated its performance improvement and parameter efficiency across multiple datasets, including cellular, nuclear, and lesion imaging tasks—directly supporting that “attention mechanisms such as CBAM enhance fine-detail recognition in microscopic and fluorescent imaging.” Hai Y [15] proposed the DEL-RESSP model, which encodes genomic alignment data as images, inputs them into a ResNet network, and integrates an attention mechanism to predict genomic deletion variants. This approach addresses the challenge of high-dimensional feature extraction in genomic data and provides a new tool for pathogenic locus identification in genetic disorders. Meanwhile, other researchers have focused on model lightweighting, transfer learning, and small-sample optimization, employing techniques such as dropout, batch normalization, and feature regularization to significantly enhance model generalization and deployment efficiency. Additionally, models that combine attention mechanisms with U-Net have demonstrated promising results in cell contour segmentation and adherent cell recognition tasks [16,17,18]. Collectively, these studies demonstrate that neural networks have achieved promising accuracy and efficiency in FISH image analysis. However, most existing approaches focus on single-modality or single-task designs (e.g., detection or segmentation) and struggle with feature fusion across multiple fluorescence channels. These limitations motivate our proposed ResNet50-based model, which integrates CBAM, PPM, and transfer learning to enhance multi-channel feature representation and classification robustness.

Despite these advances, challenges remain, including high annotation costs, class imbalance, limited model generalization, and barriers to clinical translation. Future research is expected to focus on self-supervised learning, federated learning, and explainable AI frameworks to accelerate the transition of intelligent FISH image analysis from algorithmic research to clinical and industrial applications. Based on this, this study investigates an optimized ResNet-50 network combining a CBAM and PPM to enhance multi-channel fluorescence feature extraction and multi-scale information fusion. Furthermore, transfer learning and the focal loss function are adopted to address small-sample and class imbalance issues, aiming to develop an efficient, accurate, and clinically deployable FISH image intelligent classification model.

The main contributions of this study are summarized as follows:

1. An enhanced ResNet50-based architecture is proposed for intelligent classification of FISH tissue and cell images, addressing the inefficiency and subjectivity of manual interpretation through deep residual learning.
2. A dual-attention and multi-scale fusion mechanism is designed by embedding the CBAM and PPM into residual blocks, effectively enhancing salient fluorescence feature representation and improving the detection of small or complex genomic targets.
3. A robust optimization strategy combining transfer learning and focal loss is developed to handle limited annotated samples and class imbalance, significantly boosting model accuracy and generalization.

## 2. Research Background

### 2.1. Fluorescence in Situ Hybridization

FISH, developed in the late 1970s, is a molecular cytogenetic technique that evolved from isotopic in situ hybridization [19]. It enables the visualization of specific genes or chromosomal regions within cells or tissues by using fluorescently labeled nucleic acid probes that hybridize specifically to target DNA or RNA sequences. This approach allows for the in situ detection of the position and copy number of target genes or chromosomes.

FISH technology employs different fluorescent labels to specifically mark target DNA or RNA sequences, resulting in distinctive multi-channel fluorescence characteristics in the acquired images. In practice, two to five fluorescent probes of different colors are typically used, each corresponding to distinct biological targets such as chromosomal centromeres, gene loci, or pathogen sequences. These fluorescence signals are superimposed within the same image plane, with each channel carrying independent biological information—for instance, green fluorescence may indicate the presence of a specific gene, whereas red fluorescence may represent chromosomal copy number variations.

While the multi-channel nature of FISH provides rich diagnostic information, it also substantially increases image complexity. Factors such as probe hybridization efficiency, fluorophore decay, and microscope exposure parameters can cause significant variability in signal intensity within the same image. As a result, both strong signals (e.g., amplified regions) and weak signals (e.g., single-copy genes) may coexist. Additionally, chromosomal breakage, overlap, or nonspecific binding can produce abnormal signal morphologies (e.g., trailing or dumbbell-shaped signals), which may interfere with signal counting and localization, ultimately affecting the accuracy of signal separation and recognition.

### 2.2. Target Genes

In this study, the XL RB1/DLEU/LAMP probe was employed as a qualitative, non-automated detection method for identifying deletions at the 13q14.2 RB1 gene region and the 13q14.2 DLEU1/MIR15A/MIR16-1 gene region through FISH. The 13q34 LAMP1 gene region was included as a reference locus. This probe set is designed to serve as an auxiliary diagnostic tool and to assist in disease monitoring.

**Figure 1:**
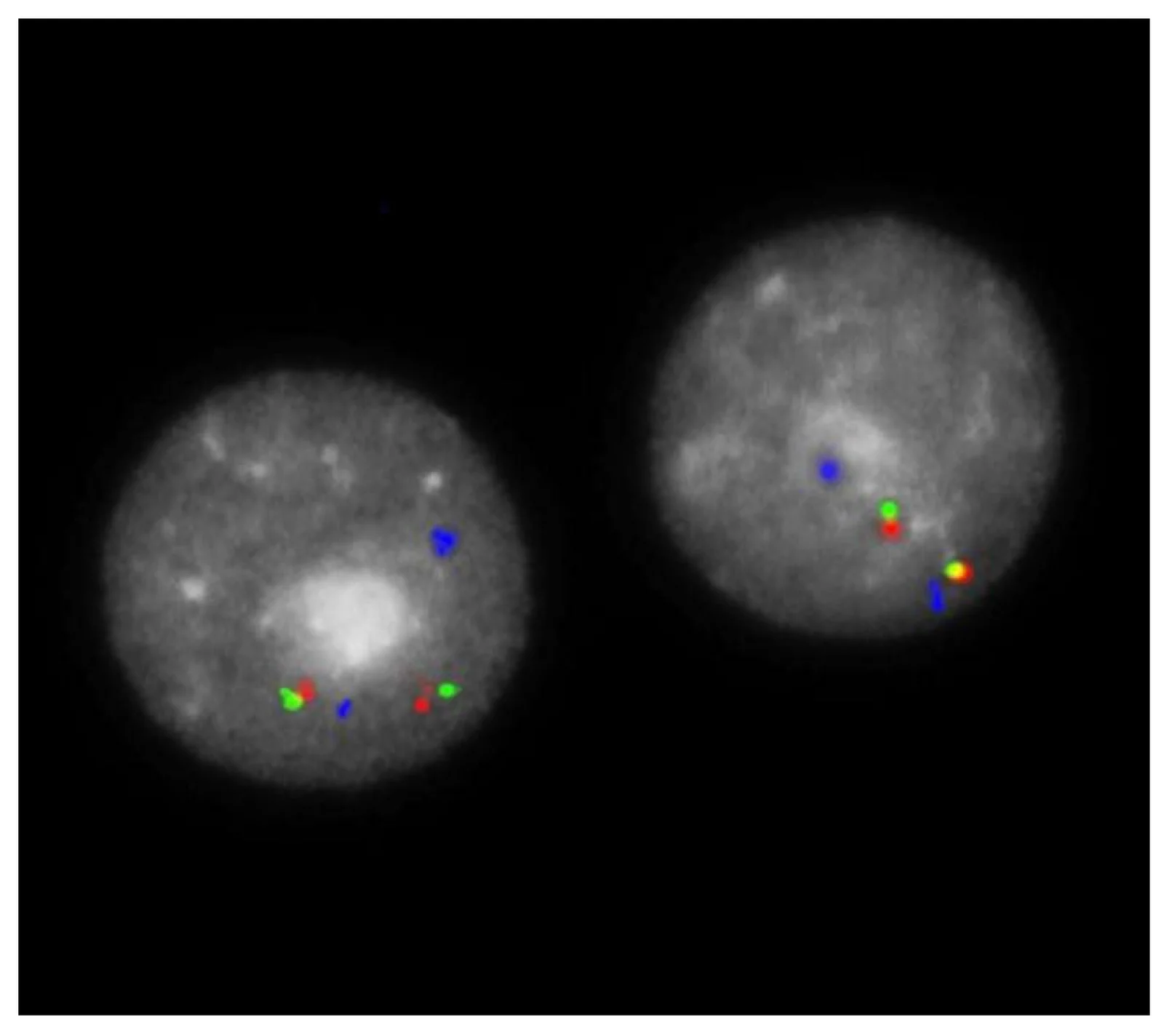
Lymphocytes hybridized with the XL RB1/DLEU/LAMP probe.

**Figure 2:**
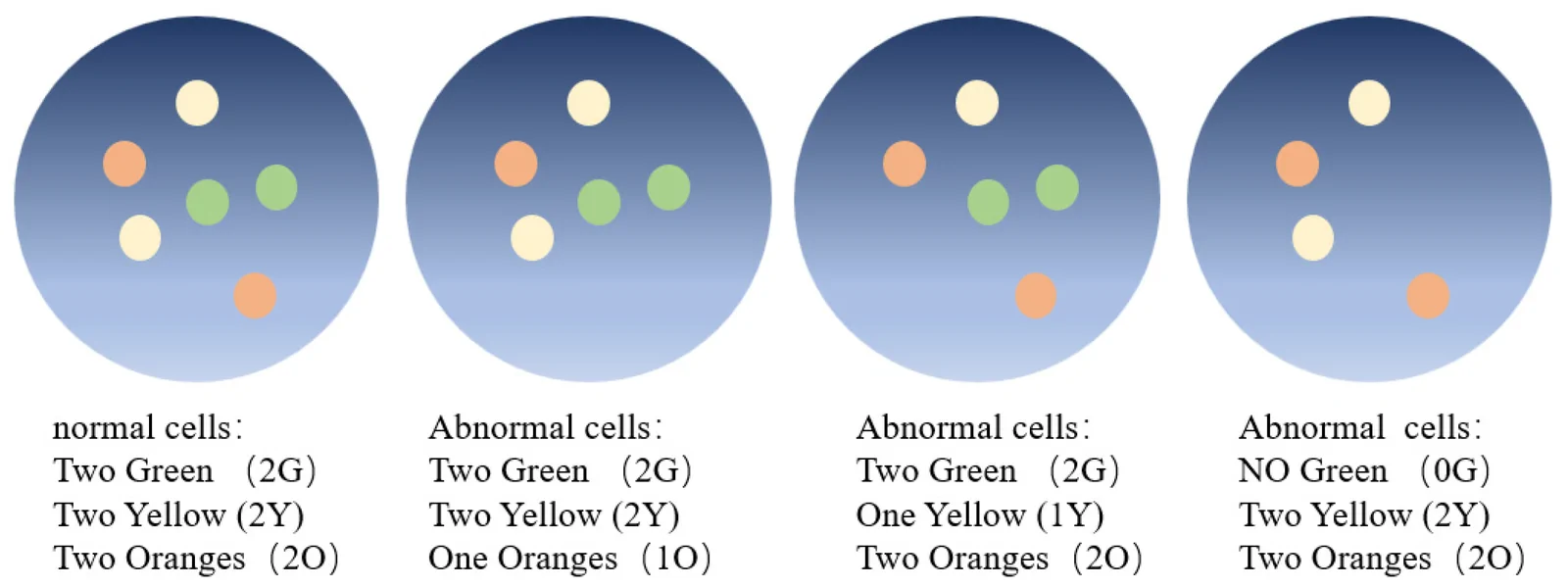
Schematic diagram of probe detection results.

The XL RB1/DLEU/LAMP probe set consists of three differently labeled probes: a green-labeled probe hybridizing to the RB1 gene region at 13q14.2, an orange-labeled probe targeting the DLEU1/MIR15A/MIR16-1 gene region (including D13S319), and an aqua-labeled probe hybridizing to the LAMP1 gene region at 13q34. This probe combination is used for the detection of 13q14 deletions and associated gene abnormalities. As shown in Figure 1 and Figure 2, among them, normal cells are Two Yellow, Two Green, and Two Orange, while the other lost cells are all abnormal cells.

### 2.3. ResNet-50 Residual Module

ResNet50 was chosen as the backbone due to its strong capability in hierarchical feature extraction and stable optimization in deep networks. The residual learning structure effectively mitigates gradient vanishing, enabling accurate modeling of complex multi-channel fluorescence textures and chromosomal morphology in FISH images. Furthermore, its modular design supports seamless integration with attention and pooling modules, making it particularly suitable for medical image classification tasks that require fine-grained feature discrimination.

ResNet (Residual Network) is a deep convolutional neural network architecture proposed by Kaiming He et al. from Microsoft Research in 2015 [20]. By introducing residual blocks, ResNet effectively addresses the problems of gradient vanishing and network degradation that occur when the network depth increases. This innovation enables the construction of networks with more than 100 layers and achieves outstanding performance in image classification and other computer vision tasks [21,22].

(1) $$y=F(x,W_{i})+x$$

In Equation (1), *x* and *y* represent the input and output features of the model, respectively; F(⋅) denotes the residual mapping $W_{i}$; $F=W_{2}\sigma (W_{1}x)$ represents the weight parameters; and σ is the activation function ReLU. The concept of Equation (1) originates from the assumption that the expected output of the underlying features is h(x)=F. By introducing a residual function F to approximate the original target function, the mapping relationship is transformed into h(x)−x. When the residual equals zero, the stacked layers perform at least an identity mapping; however, in practice, the residual is nonzero, ensuring that the network performance does not degrade.

By introducing the residual module, the network can connect shallow and deep feature representations through a “shortcut” mechanism, effectively enhancing the overall performance [23]. The ResNet family includes various configurations with different depths, such as ResNet-18, ResNet-34 [24], ResNet-50 [25], ResNet-101 [26], and ResNet-152 [27].

**Figure 3:**
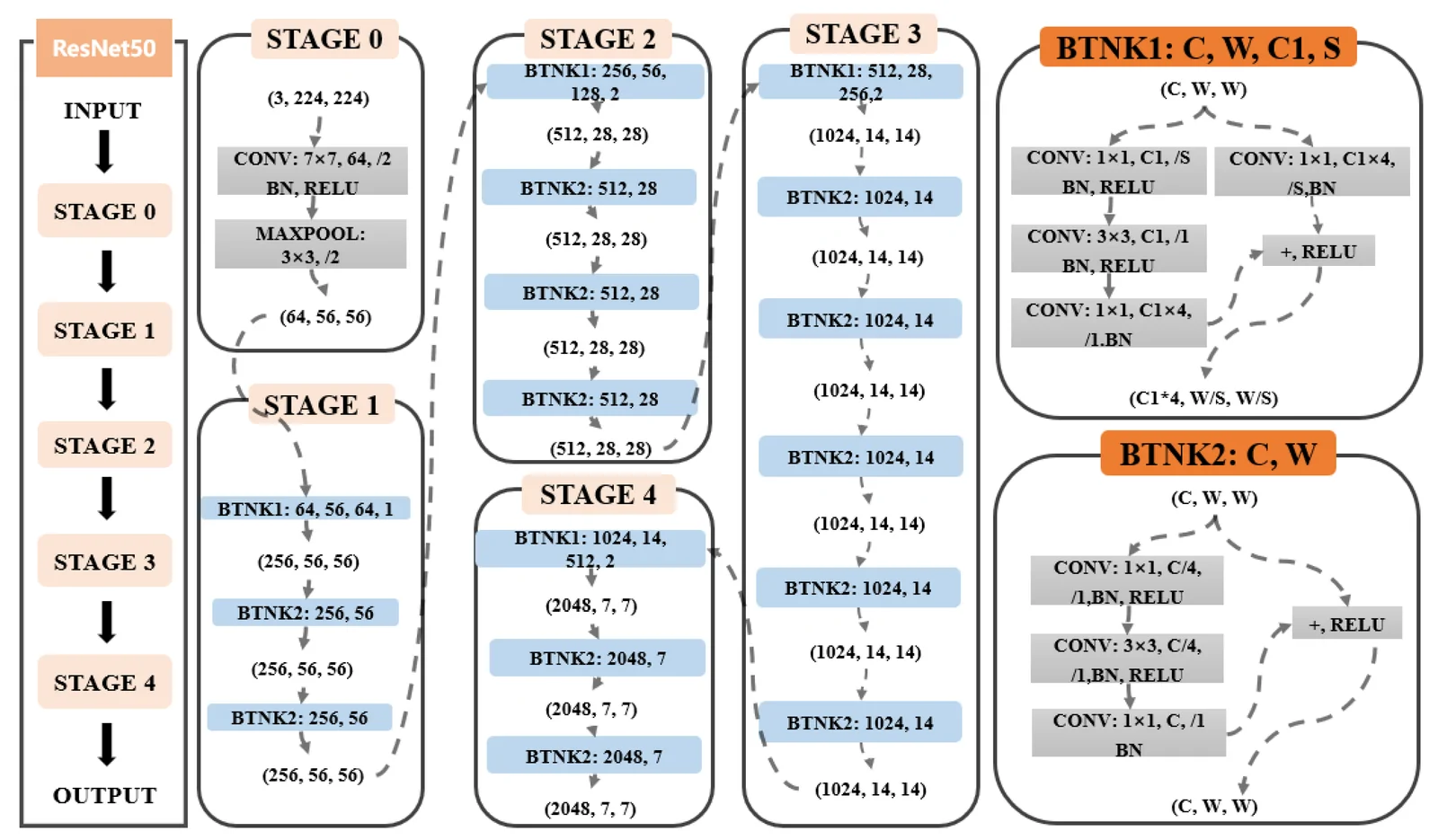
ResNet-50 processing flow.

ResNet-50 [28,29] is a deep convolutional neural network based on the residual network (ResNet) architecture, as shown in Figure 3. Its core idea is to address the vanishing gradient problem in deep networks through residual modules and skip connections, thereby enabling the training of much deeper models.

## 3. Optimization of ResNet-50 Model Based on CBAM-PPM

### 3.1. Integration of CBAM

To address the multi-channel fluorescence signals and complex spatial distribution characteristics present in FISH images, a CBAM was embedded after each residual block of the ResNet-50 architecture. In the ResNet-50 backbone, CBAMs are integrated into each residual unit to enhance the feature representation capability through a dual-path attention mechanism.

**Figure 4:**
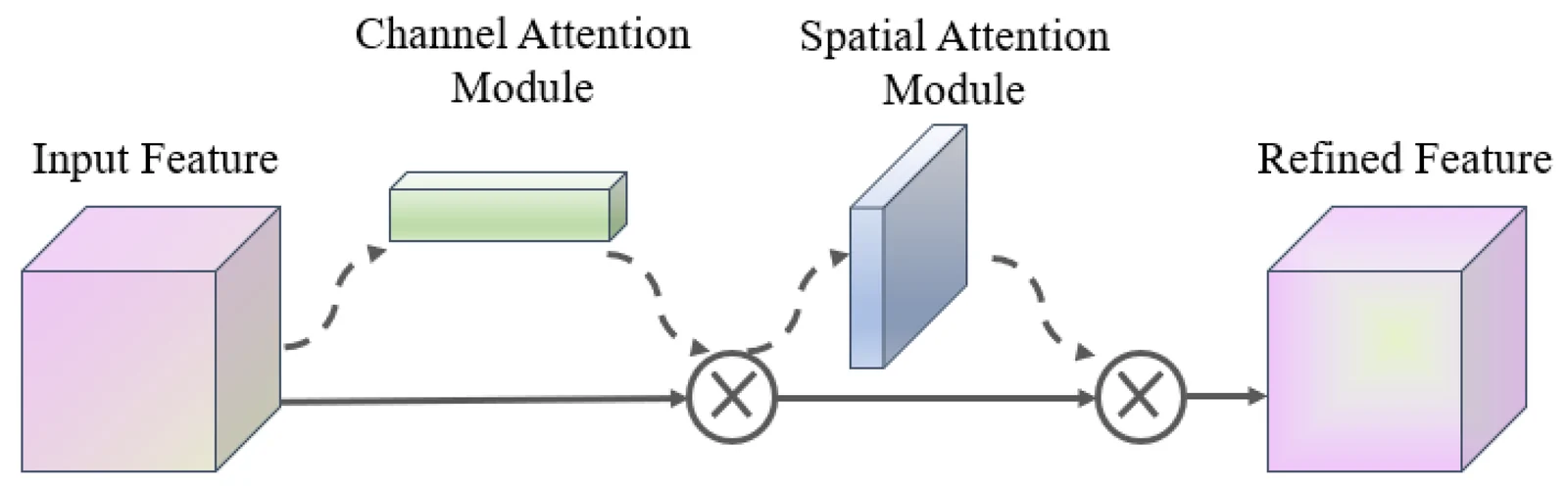
Structure of the CBAM.

This hybrid attention mechanism consists of two complementary submodules—Channel Attention and Spatial Attention—which jointly refine the feature maps by adaptively focusing on informative regions while suppressing irrelevant background responses, as illustrated in Figure 4.

In the channel attention branch, the feature maps are simultaneously processed by global average pooling (GAP) and global max pooling (GMP) layers to extract channel-wise statistical information. The two pooled feature vectors are then passed through a shared multilayer perceptron (MLP) for nonlinear transformation, and subsequently fused to generate the channel attention weight matrix. This mechanism enables dynamic optimization of fluorescence channel representations (e.g., Cy3, FITC, DAPI), and effectively suppresses non-specific fluorescence interference, such as autofluorescence signals from the cytoplasm.

In the spatial attention branch, a 7 × 7 convolution kernel with a large receptive field is applied to construct a spatial attention map, thereby enhancing the model’s sensitivity to subcellular localization. Through spatial weighting, the network amplifies the representation strength of signal-dense regions (such as punctate clusters around the nuclear membrane) while attenuating background noise from cytoplasmic regions. This spatial selection mechanism preserves the topological correlation between fluorescently labeled loci and cellular morphological features.

**Figure 5:**
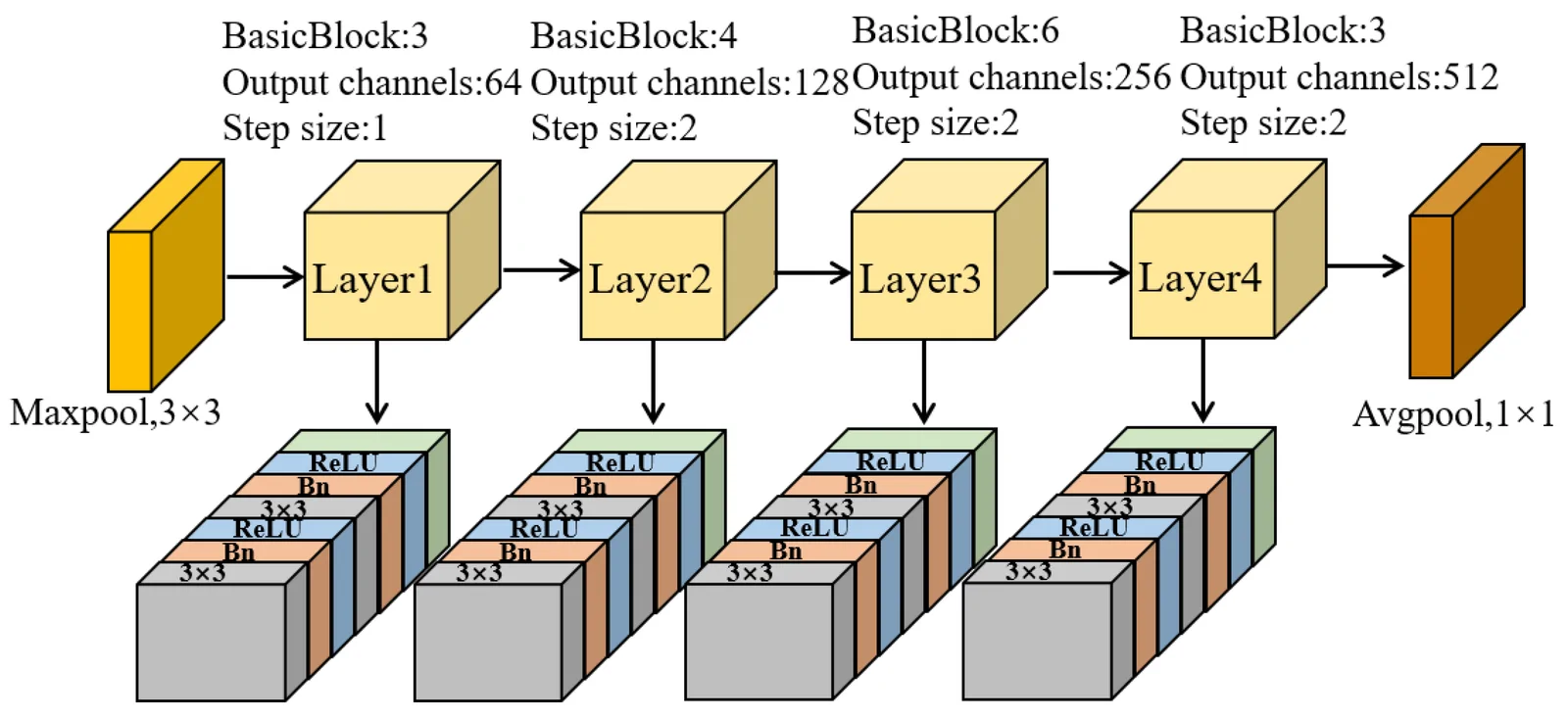
Hierarchical Localization Map.

Within the ResNet-50 architecture, CBAMs are embedded at the output of each residual block, immediately after the final ReLU activation layer, serving as the terminal feature refinement processor for each block, as shown in Figure 5. In total, 16 CBAMs are integrated across all four stages (Conv2_x to Conv5_x) of the network, as detailed below:

Conv2_x stage: 3 residual blocks, each followed by one CBAM.

Conv3_x stage: 4 residual blocks, each followed by one CBAM.

Conv4_x stage: 6 residual blocks, each followed by one CBAM.

Conv5_x stage: 3 residual blocks, each followed by one CBAM.

### 3.2. Integration of PPM

To address the multi-scale distribution of fluorescent signals in FISH images (e.g., the size differences between single-copy signals and clustered signals), a \PPM\ was integrated at the output of the Conv5_x stage of the ResNet50 backbone. This architecture significantly enhances the capture of multi-scale contextual information through a hierarchical feature extraction mechanism.

**Figure 6:**
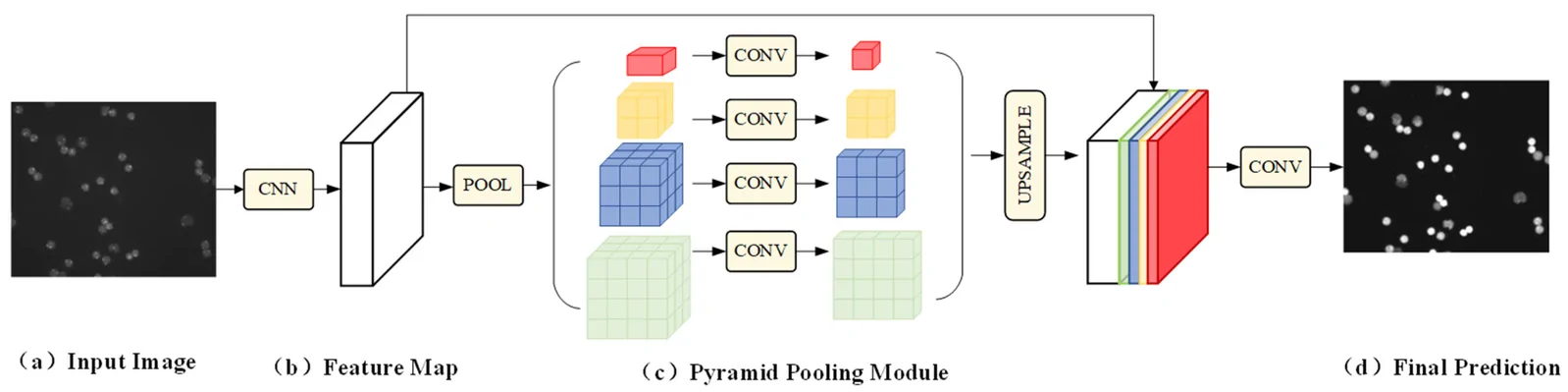
PPM Architecture.

The PPM employs a staged pooling strategy for multi-granularity feature analysis: the first level applies 1 × 1 global pooling to obtain statistical representations of signal distribution across the entire field of view (e.g., signal density gradients and overall intensity distribution); the second level uses 2 × 2 grid pooling to analyze subcellular-scale signal cluster morphology, including inter-signal spacing and spatial arrangement topology; the third level performs 4 × 4 grid pooling to focus on fine-grained local structural features, such as gradient variations at the edges of micro-deficient signals. Through bilinear interpolation upsampling and channel-wise concatenation, features at different scales are fused into a composite representation vector rich in spatial semantic information. As shown in Figure 6.

This hierarchical feature fusion mechanism exhibits two main technical advantages. First, the global contextual information provides a biological reference for the classification of local signals. Second, the collaborative effect of multi-resolution features effectively addresses the problem of small-scale targets (e.g., micro-deficient signals) being easily overwhelmed by background noise in conventional convolutional networks.

Within the ResNet50 backbone, the PPM is precisely embedded at the output of the Conv5_x (layer4) stage. The 7 × 7 × 2048 feature map generated at this stage serves as the input to the PPM. After processing by the PPM, a 7 × 7 × 2560 feature map is produced, which is directly connected to the downstream Global Average Pooling layer, providing enhanced feature representations for subsequent classification or detection tasks.

The PPM adopts an innovative four-branch parallel structure to process the input features, as detailed in Table 1.

The outputs of the four branches are concatenated along the channel dimension, resulting in a 7 × 7 × 2560 feature map (1024 + 512 × 3 channels) that integrates multi-scale information. This enhanced feature map encompasses multi-dimensional information ranging from global macro context to local microstructures, providing more discriminative representations for subsequent tasks.

### 3.3. Incorporation of Transfer Learning

#### 3.3.1. Implementation Procedure

To address the scarcity of annotated FISH images, a two-stage transfer learning strategy was employed to optimize the model:

(1) Pre-training Stage

The ResNet50 backbone was trained on the dataset we have built to learn generic image features, including low-level visual features (edges, textures, shapes) and high-level semantic features (object parts, category semantics). This pre-training endows the model with fundamental feature extraction capabilities, reducing its dependence on the limited FISH annotation data.

(2) Fine-tuning Stage

The pre-trained ResNet50 model was transferred to the FISH image classification task, and network parameters were adjusted for the specific scenario. Low-level features learned from pre-training were reused, while only high-level classification layers and certain mid-level features were adaptively updated.

#### 3.3.2. Key Optimization Strategies

(1) Layer-wise Parameter Freezing

Freeze the first two convolutional layers: The initial layers (Conv1 and Conv2_x) primarily extract low-level visual features (edges, colors, textures) that are highly consistent between generic images and FISH images. Freezing these parameters prevents overfitting or degradation of low-level features due to the limited FISH dataset.

Update high-level network parameters: Starting from Conv3_x, high-level layers extract semantic features (e.g., cell morphology, signal distribution patterns). Parameters in these layers are left trainable to learn task-specific features, such as the morphology of fluorescent signals.

(2) Fully Connected Layer Reconstruction and Classification Adaptation

Replace the output layer: The original 1000-dimensional fully connected layer (for ImageNet classification) was replaced with a 5-dimensional layer to predict the five FISH image categories (normal, trisomy, deletion, translocation, fusion).

Loss function and optimization objective: Cross-entropy loss was employed to measure the discrepancy between predicted labels and ground truth, with the optimization objective of minimizing this loss. As shown in Equation (2).

(2) $$L=-\sum_{i=1}^{N}\sum_{c=1}^{5}y_{i,c}\log{\hat{y}}_{i,c}$$

Here, *N* denotes the number of samples, $y_{i,c}$ represents the ground-truth label (0 or 1), and ${\hat{y}}_{i,c}$ corresponds to the predicted class probability output by the model.

(3) Learning Rate Decay Strategy

The initial learning rate was set to a moderate value to prevent drastic fluctuations of the pre-trained parameters. After every 5 epochs (i.e., one full pass through the dataset), the learning rate was multiplied by a decay factor of 0.5. As shown in Equation (3).

(3) $$Ir=Ir_{initial}\times {\left(0.5\right)}^{\frac{epoch}{5}}$$

In the early stages, a relatively high learning rate accelerates the convergence of the newly added classification layers. In later stages, a lower learning rate fine-tunes the high-level features, balancing the integration of old and new knowledge while mitigating overfitting.

#### 3.3.3. Experimental Results and Data Comparison

By leveraging the general features of the pre-trained model, transfer learning significantly reduces the dependence of FISH image analysis on large-scale annotated datasets. The combination of layer-wise freezing and learning rate decay effectively balances the need to retain pre-trained knowledge and adapt to the new task, enhancing model stability and generalization under data-scarce scenarios, as summarized in Table 2.

Transfer learning demonstrates significant advantages in FISH image analysis tasks, particularly in data-scarce scenarios. The transfer learning model requires only 50% of annotated data to accomplish the task, compared with the baseline model, which depends on the full dataset. This represents a 100% improvement in data efficiency, effectively alleviating the high cost and difficulty of large-scale manual annotation in FISH image analysis.

In small-sample scenarios, the transfer learning model converges in only 14 epochs, 6 epochs fewer than the 20 epochs required by the baseline model, representing a 30% acceleration in convergence speed. This reduction in training time decreases computational resource consumption, making it particularly suitable for real-time or rapid analysis applications.

The transfer learning model achieves a generalization error of 22.9% on the test set, compared to 28.5% for the baseline, indicating higher predictive accuracy on unseen samples. By employing layer-wise parameter freezing and learning rate decay strategies, the model retains pre-trained knowledge while adapting to the new task, avoiding overfitting or underfitting. This approach is particularly robust under limited data conditions. By balancing retention of pre-trained knowledge and adaptation to new tasks, it addresses common transfer learning issues such as catastrophic forgetting of old knowledge or insufficient learning of new information, serving as a key factor in enhancing model performance.

Overall, the combination of transfer learning with layer-wise freezing and learning rate decay provides an efficient, low-data-reliance, and high-generalization solution for FISH image analysis. This approach is especially valuable in biomedical applications where annotation costs are high and sample sizes are limited, demonstrating substantial practical significance and strong potential for broader adoption.

## 4. Evaluation Indexes

**Figure 7:**
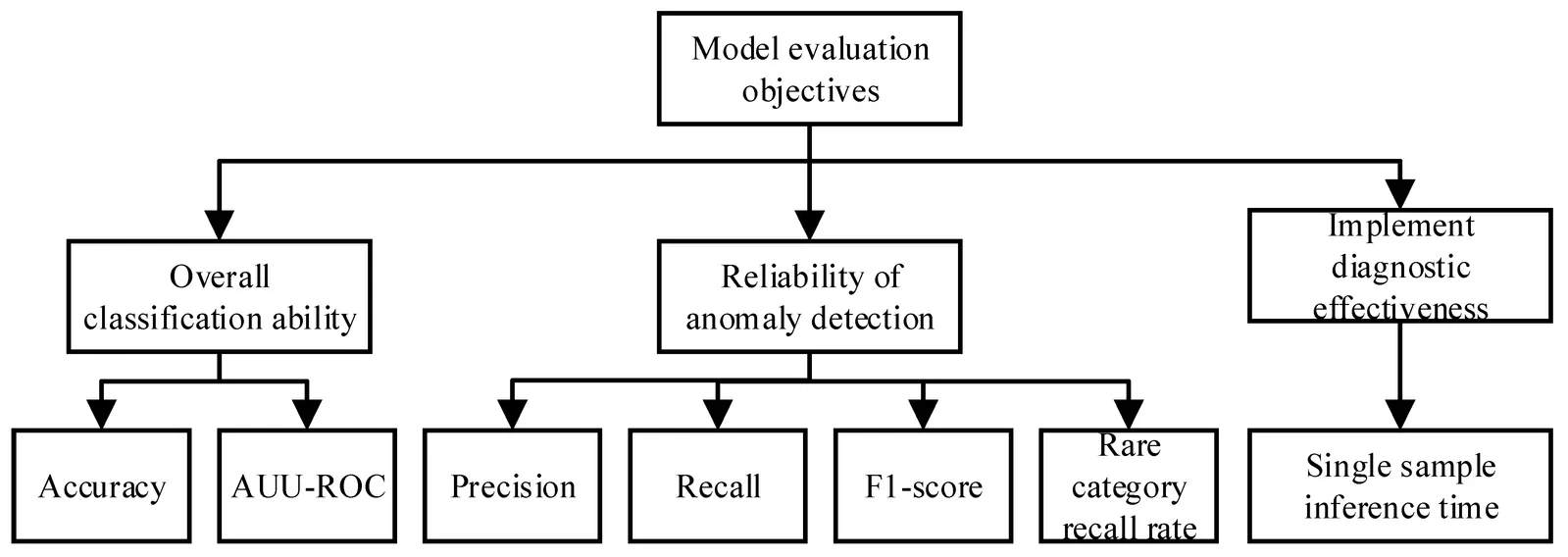
Evaluation Framework.

This evaluation system is designed based on the clinical requirements for FISH chromosomal abnormality classification, establishing a three-tier assessment framework encompassing overall performance, abnormality detection, and real-time diagnosis. By performing cross-validation across multiple metrics, the framework ensures the reliability and practicality of the model in real-world applications, as illustrated in Figure 7.

(1) Overall Performance Metrics

Accuracy: Reflects the model’s classification correctness across the entire sample space, representing its fundamental recognition capability, as shown in Equation (4).

(4) $$Accuracy=\frac{TP+TN}{TP+TN+FP+FN}$$

Area Under the Receiver Operating Characteristic Curve (AUC-ROC): Provides a comprehensive measure of the model’s generalization ability across different classification thresholds and demonstrates robustness to class imbalance, as shown in Equation (5).

(5) $$AUC=\int_{0}^{1}TPR(FPR)dFPR$$

(2) Abnormality Detection-Specific Metrics

Precision: Also known as positive predictive value, measures the accuracy of the model in identifying positive cases, helping to avoid overdiagnosis and the associated psychological burden on patients, as shown in Equation (6).

(6) $$P=\frac{TP}{TP+FP}$$

Recall: Measures the model’s ability to capture true positive cases, enhancing detection of high-risk instances and preventing missed diagnoses, as shown in Equation (7).

(7) $$R=\frac{TP}{TP+FN}$$

F1-score: The harmonic mean of precision and recall, balancing the effects of class imbalance between positive and negative samples, as shown in Equation (8).

(8) $$F1=2\times \frac{P\times R}{P+R}$$

Note: TP= True Positives, FP= False Positives, TN= True Negatives, FN= False Negatives.

(3) Real-Time Diagnostic Performance Metrics

In clinical diagnostic scenarios, the algorithm’s real-time responsiveness directly affects diagnostic efficiency and workflow adaptability. The core focus is the end-to-end inference time per single sample, defined and evaluated as follows:

The metric encompasses the complete processing pipeline, including image preprocessing (e.g., grayscale correction, noise suppression, and size normalization), model forward inference computation (feature extraction and classification prediction), and post-processing (result parsing and visualization output), with time measured in milliseconds (ms). The metric must meet clinical requirements: inference time per single sample ≤ 50 ms, supporting the diagnosis of 20 samples per second in batch mode. Moderate increases in processing time (≤200 ms) are permissible, provided interaction fluency is maintained.

## 5. Experimental Results and Analysis

### 5.1. Experimental Setup

#### 5.1.1. Experimental Data

The study utilized a clinically collected FISH image dataset containing two classes of cell images (good, bad), totaling 12,000 images. The dataset was split as follows: 8000 images for training (including 2000 GAN-generated samples), 2000 for validation, and 2000 for testing.

Data augmentation strategies were applied to simulate varying cell orientations and imaging conditions: random rotations (±15°), horizontal/vertical flips (*p* = 0.5), and random cropping to 224 × 224 pixels simulated diverse spatial distributions on slides. Brightness (±20%), contrast (±15%), and saturation (±10%) adjustments simulated differences in illumination across imaging devices.

#### 5.1.2. Hardware and Software Environment

Training and testing were conducted under the following environment: Windows 11 OS, PyCharm IDE, Python 3.9. The system was equipped with an AMD Ryzen 9 7945HX CPU and an NVIDIA RTX 4060 GPU (8 GB VRAM). GPU acceleration was preferred for model training, with CPU training supported at slower speeds. The deep learning framework used was PyTorch 2.0, enabling dynamic computation graph-based model development and efficient debugging.

#### 5.1.3. Training Parameters

The training process employed carefully tuned hyperparameters to balance model performance and training efficiency. As shown in Table 3.

### 5.2. Results and Analysis

#### 5.2.1. Classification Performance

**Figure 8:**
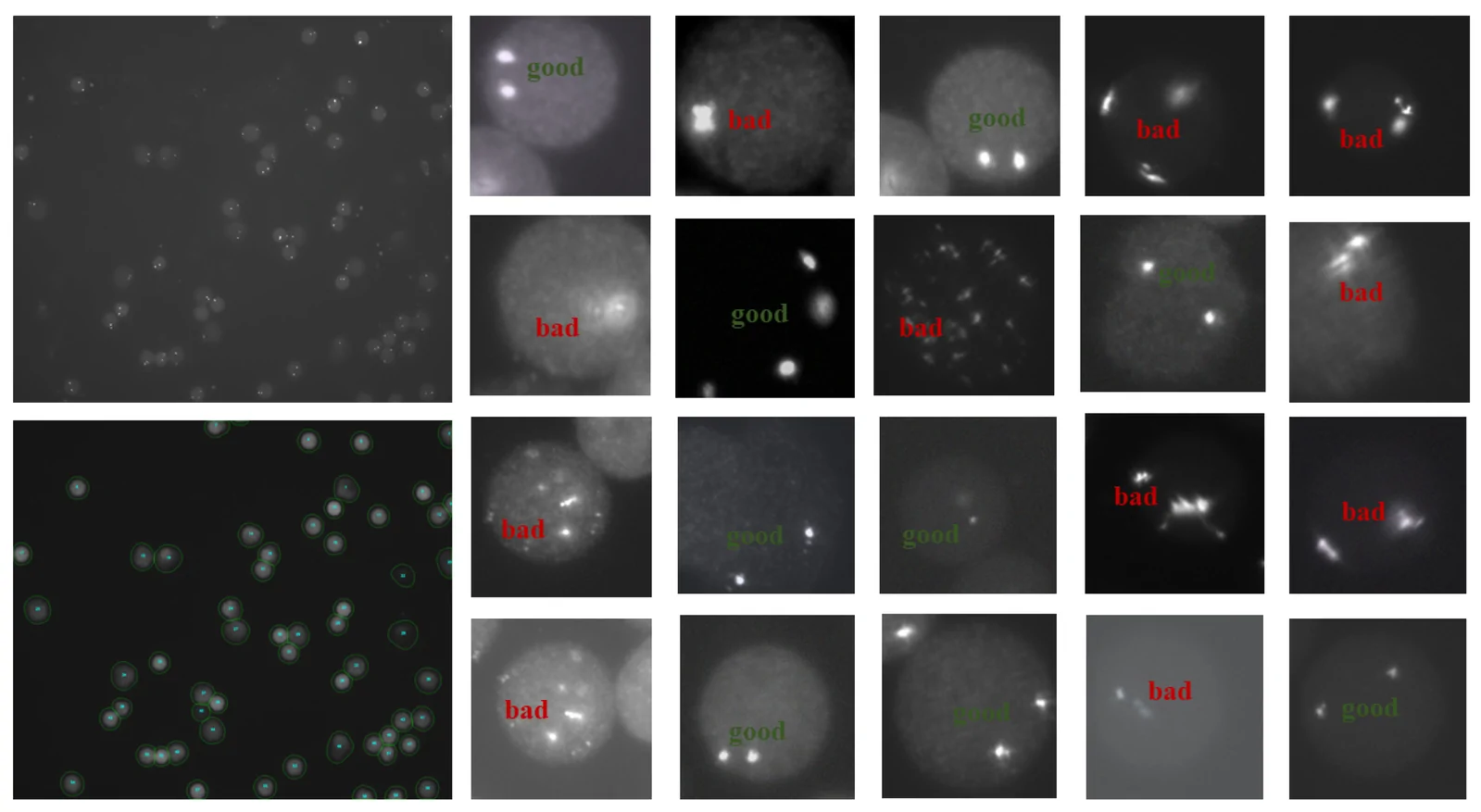
Cell image classification results.

After execution, the program automatically selects the optimal model for classification, accurately displaying the classification results of the images, as illustrated in Figure 8. This provides an intuitive visualization of the model’s classification capability, clearly demonstrating its performance.

#### 5.2.2. Training Dynamics and Loss Analysis

**Figure 9:**
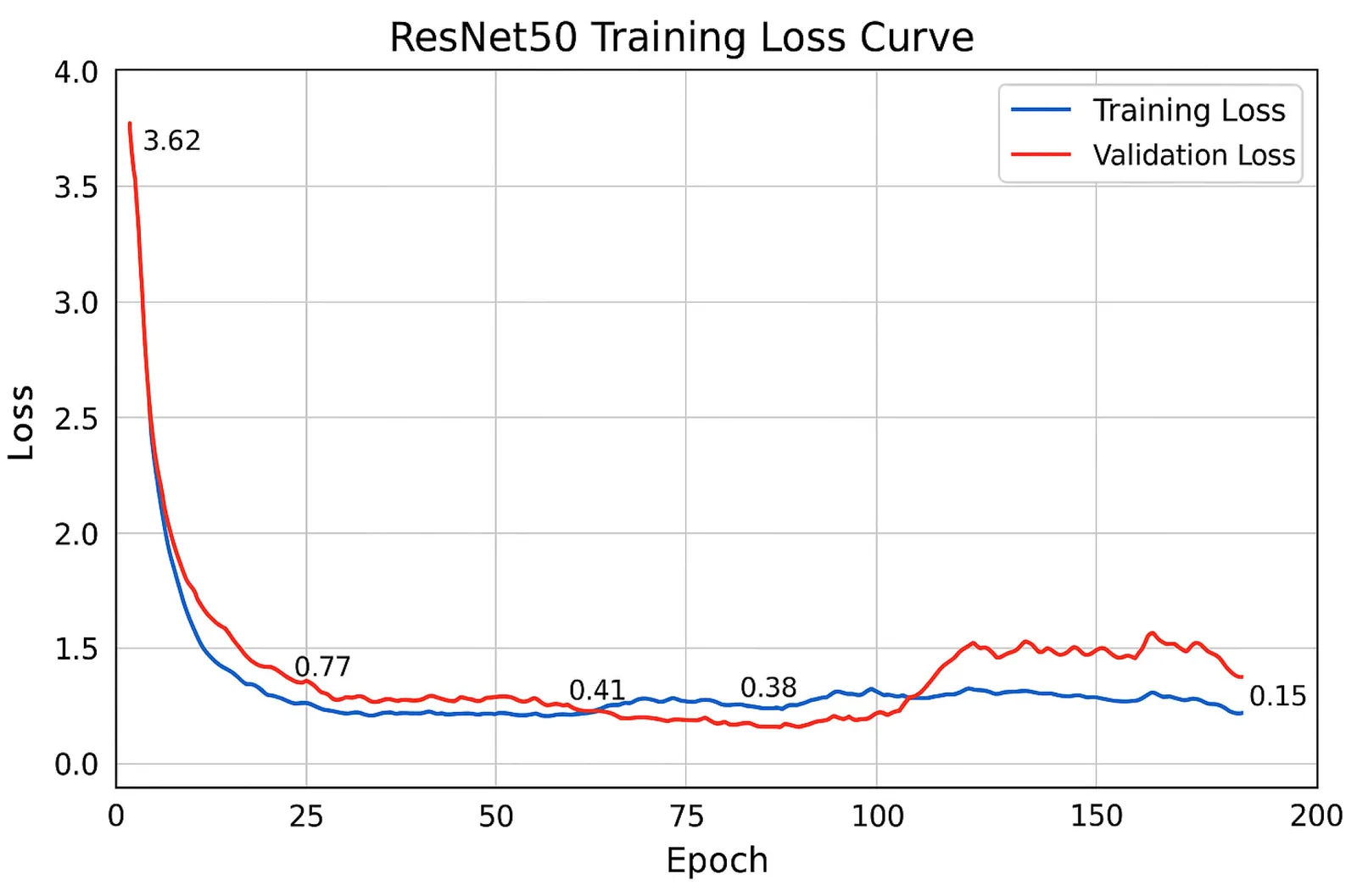
Loss Function Curves.

The performance of the conventional cross-entropy loss (CE) was compared with the improved focal loss (FL) on an imbalanced dataset, where the abnormal class accounts for 30% of the samples. Focal loss adjusts the weights of easy and hard samples (γ = 2), increasing the model’s attention to the rare abnormal class. Compared with standard cross-entropy loss, focal loss reduces the classification error on the rare class (bad) by 25.6%, significantly enhancing the model’s ability to detect abnormal signals, as illustrated in Figure 9.

**Figure 10:**
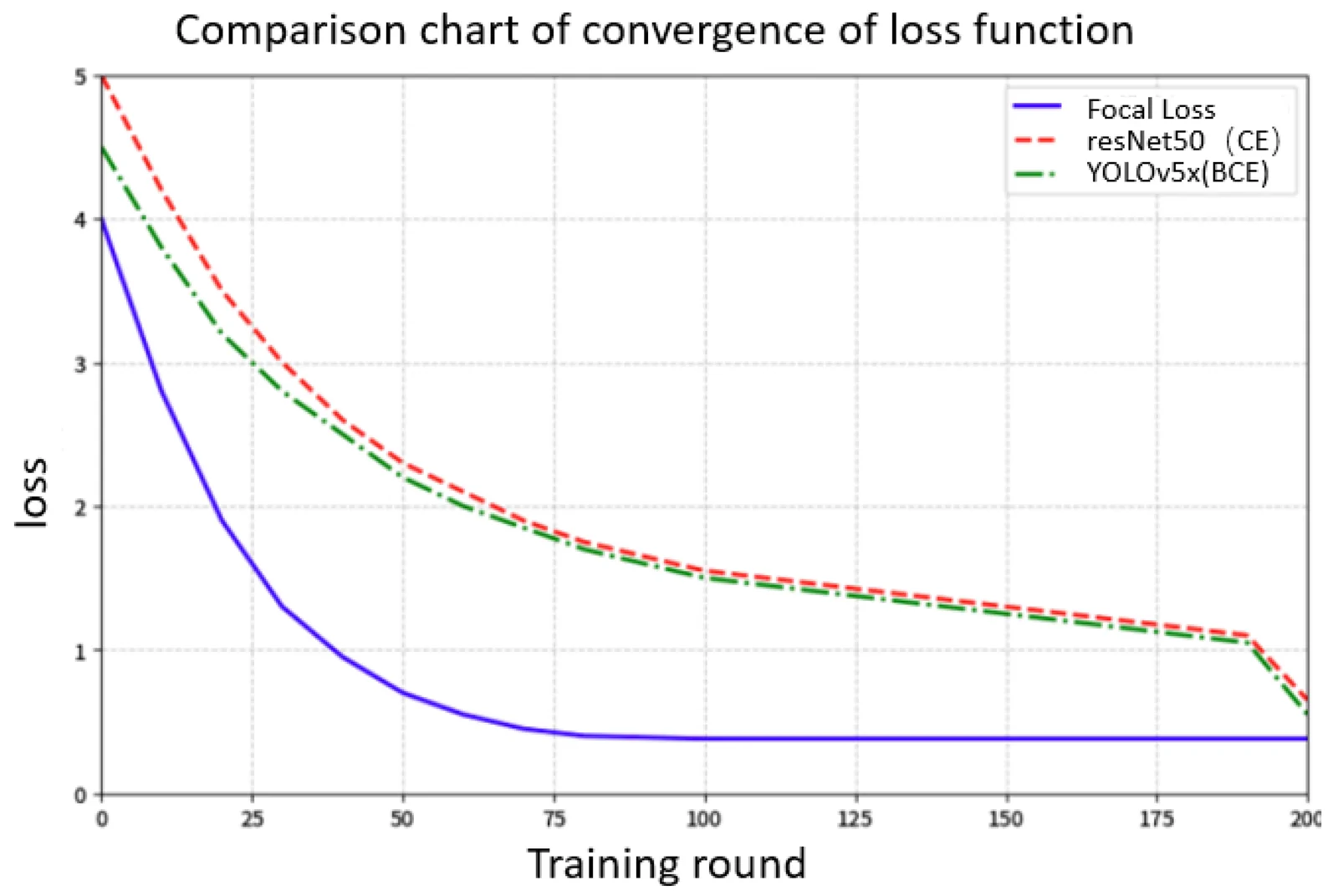
Comparison of loss function convergence among different models.

After adopting focal loss, the training loss of the proposed model converged 40% faster than ResNet50 and 20% faster than YOLOv5x. This improvement is attributed to the dynamic weighting of hard samples, preventing premature convergence to local minima. On the validation set, the model achieved a loss of only 0.15, significantly lower than other models (ResNet50 series > 0.55), indicating superior fitting of the complex distribution of FISH images. On the dataset with a 30% proportion of abnormal classes, focal loss reduces the weight of easily classified samples (α = 0.5, γ = 2), decreasing the classification error for rare classes (e.g., translocation, fusion) by 25.6%. In contrast, models trained with conventional cross-entropy loss exhibited frequent missed diagnoses, as illustrated in Figure 10.

**Figure 11:**
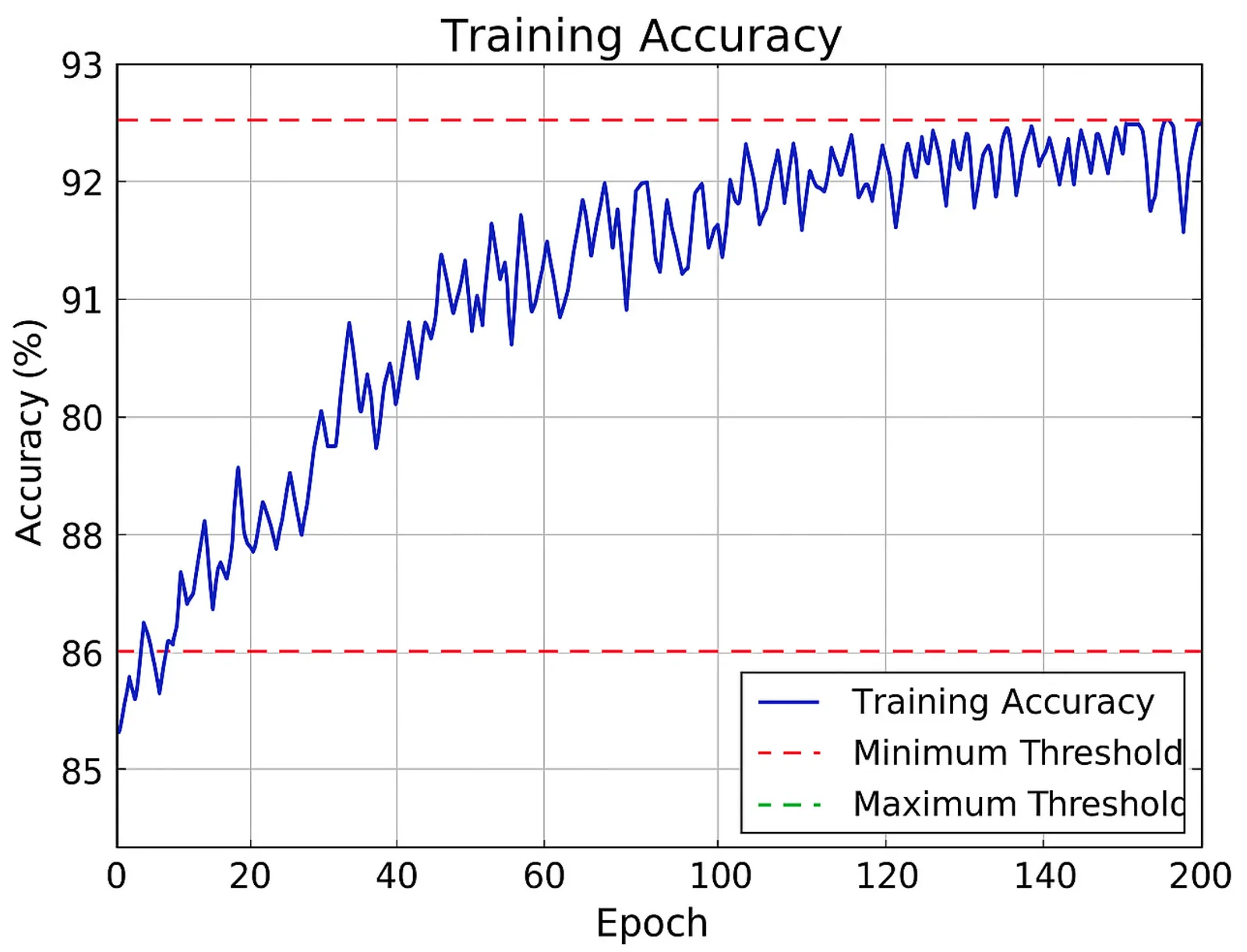
Training Accuracy vs. Epochs.

The model stabilized after 110 epochs, with the validation accuracy reaching its peak of 92.4% at the 123rd epoch and remaining steady thereafter. The ReduceLROnPlateau strategy was employed to dynamically adjust the learning rate, resulting in consistent accuracy across ten test sets, with a maximum of 92.4%, as illustrated in Figure 11.

**Figure 12:**
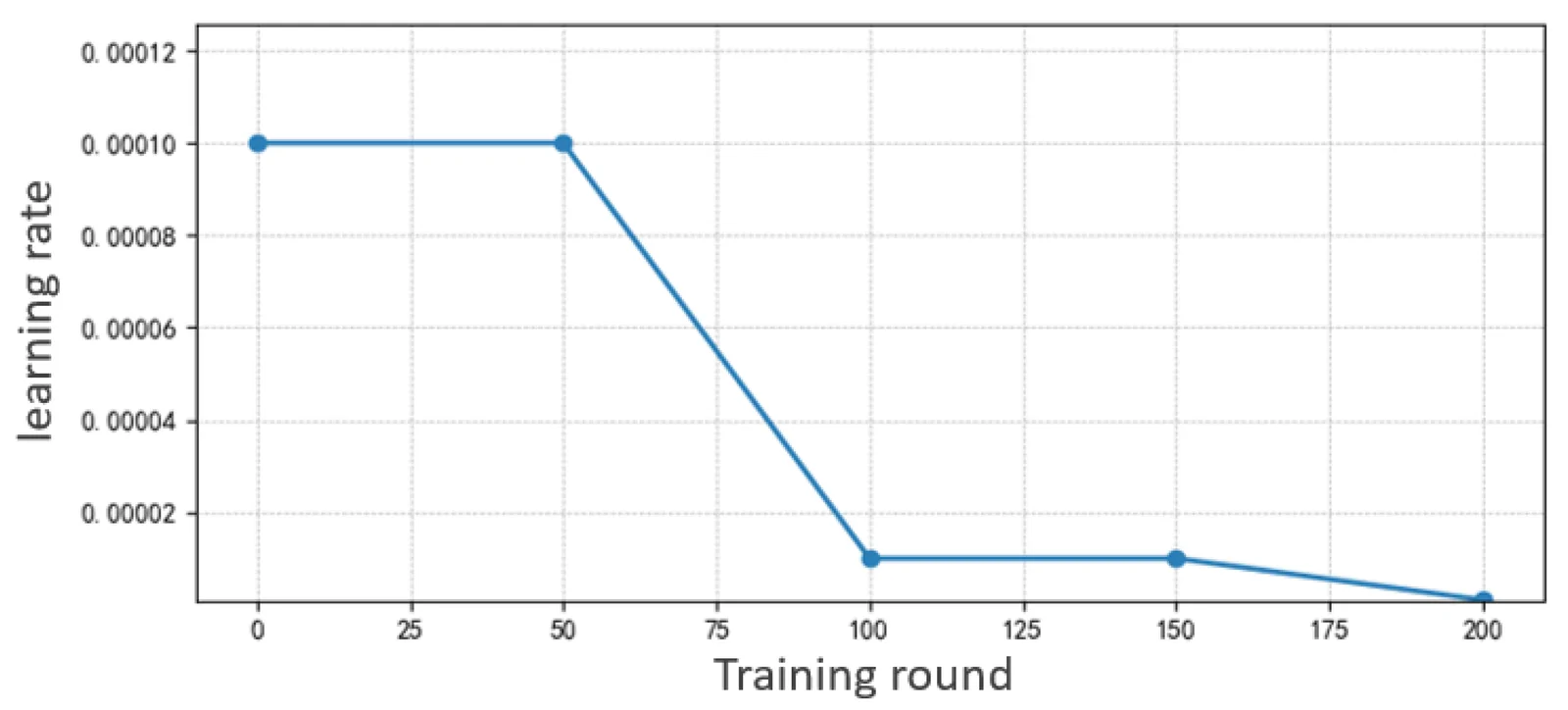
Learning rate variation curve under the Reduce LROn Plateau strategy.

Using this strategy, the learning rate gradually decreased from $10^{-4}$ to $10^{-6}$, effectively balancing model convergence speed and accuracy, as illustrated in Figure 12.

#### 5.2.3. Ablation Experiments

Ablation experiments were conducted to evaluate the contributions of each optimization component, with results summarized in Table 4.

Transfer learning contributed the most (+6.1%), highlighting the effectiveness of pre-trained models in small-sample scenarios. CBAM and PPM exhibited a synergistic effect, with their combined usage outperforming individual application. The two-stage training strategy (freezing + fine-tuning) accelerated convergence by 35%, while data augmentation mitigated overfitting, resulting in a 28% reduction in validation loss.

#### 5.2.4. Comparison of Different Network Architectures

The performance of various network architectures was compared on the test set, including Faster R-CNN + FPN, YOLOv4, YOLOv5x, YOLOv7, SSD513, DSSD513, DETR, ViT-FRCNN, ViDT, and the proposed optimized model (ResNet50 + CBAM + PPM + Transfer Learning). The results are summarized in Table 5.

The comparison results of multiple models are presented in Table 6. The proposed optimized model achieved a classification accuracy of 92.4%, significantly outperforming YOLO series, DETR, and ViT-based models. The inference time was 22.3 ms per sample, meeting the clinical requirement of ≤50 ms per sample, and the recall rate for complex classes exceeded 90.7%. Compared with the baseline results in Table 7, CBAM improved accuracy by 4.8%, PPM improved accuracy by 3.4%, and the fully optimized model improved accuracy by 9.9% over the original ResNet50. Although the inference time increased by 19.3%, it remained within an acceptable range for practical use.

The final optimized model achieved an accuracy of 92.5% on the good class and 92.3% on the bad class. The adoption of focal loss resulted in an F1-score of 91.1% for the bad class, and the batch processing throughput reached 1432 samples per second.

This study conducted comprehensive experimental validation on the proposed ResNet50-based optimized classification model for FISH tissue cell images. Through multi-dimensional comparison and analysis, the model’s accuracy, robustness, and clinical applicability were systematically verified.

1. The optimized model, trained on 12,000 clinical FISH images, integrates multi-scale feature fusion (PPM) and channel–spatial attention (CBAM) modules, supported by transfer learning and focal loss strategies. Experimental results demonstrate that the model achieves an overall accuracy of 92.4%, outperforming the baseline ResNet50 by 9.9%. Particularly for complex abnormal categories (e.g., 13q14.2 deletion and fusion), the recall rate exceeded 90.7%, and the AUC-ROC reached 0.945, highlighting the model’s strong discriminative capability.
2. In terms of computational efficiency, the model achieves 22.3 ms per sample and a batch throughput of 1432 samples/s, meeting real-time diagnostic requirements and significantly surpassing Faster R-CNN and YOLOv7 in inference speed. The ablation analysis revealed that transfer learning contributed the largest performance gain (+6.1%), while the joint use of CBAM and PPM yielded a synergistic enhancement of 8.2%. Additionally, focal loss effectively mitigated data imbalance, reducing classification errors for rare categories by 25.6%.

## 6. Conclusions

This study focuses on the challenging task of classifying FISH tissue cell images, addressing limitations of traditional manual interpretation, such as low efficiency and poor consistency, as well as the intrinsic complexity of multi-channel fluorescent signals in FISH images, that pose significant challenges for clinical diagnosis. We propose a ResNet50-based optimized classification algorithm, which incorporates a CBAM, a PPM, and improved residual block shortcut connections. In addition, transfer learning, focal loss, and data augmentation strategies were employed to enhance model performance, effectively addressing critical issues such as multi-channel signal processing, small-sample training, and recognition of complex morphological patterns.

Experimental results demonstrate that the optimized model exhibits excellent performance for clinical applications. On the test set, the model achieved a classification accuracy of 92.4%, representing a 9.9% improvement over the original ResNet50, substantially enhancing FISH image classification accuracy. For rare abnormal classes, such as translocations and fusions, recall rates were significantly improved, reducing the risk of missed diagnoses and providing strong support for precise disease diagnosis. In terms of inference efficiency, the model required only 22.3 ms per sample, meeting the demands of real-time clinical diagnosis and enabling rapid screening and analysis. This performance improvement can enhance medical workflow efficiency and alleviate the workload of pathologists.

However, some limitations remain. First, the dataset was collected from a single clinical source, which may limit cross-center generalization. Second, the proposed model primarily processes 2D fluorescence images, without leveraging potential 3D spatial or temporal information that could further improve diagnostic interpretability. Third, although the CBAM–PPM integration enhances feature extraction, it slightly increases computational complexity, which may constrain deployment on resource-limited medical devices. Future work will therefore focus on multi-center data validation, lightweight model optimization, and integration with multimodal biomedical imaging to extend the method’s generalization and interpretability in broader diagnostic scenarios.

## Figures and Tables

**Table 1:** Implementation of PPM Structure.

Branch Name	Processing Pipeline	Output Dimension
Main Feature Path	1 × 1 Convolution for dimensionality reduction	7 × 7 × 1024
Global Context Branch	Global Average Pooling → 1 × 1 Convolution → Bilinear Upsampling	7 × 7 × 512
Subcellular-scale Branch	2 × 2 Grid Pooling → 1 × 1 Convolution → Bilinear Upsampling	7 × 7 × 512
Local Microstructure Branch	4 × 4 Grid Pooling → 1 × 1 Convolution → Bilinear Upsamplin	7 × 7 × 512

**Table 2:** Experimental operation results.

Metric	Baseline Model	Transfer Learning Model	Improvement
Convergence Speed in Small-Sample Scenario	20 epochs	14 epochs (30% faster)	+30%
Generalization Error (Test Set)	28.5%	22.9% (reduced by 19.7%)	−19.7%
Annotation Data Requirement	Full dataset	50% fewer annotations	2× data efficiency

**Table 3:** Training Hyperparameter Configuration.

Metric	Baseline Model
batch_size’: 32,	Batch size, controlling the number of samples processed per batch
epochs’: 200,	Number of training epochs, indicating how many times the entire dataset is traversed
lr’: 0.0001,	Initial learning rate, controlling the step size for parameter updates
weight_decay’: 1 × 10^−4^,	Weight decay to prevent overfitting
dropout_rate’: 0.5,	Dropout rate to suppress overfitting
loss_type’: ‘focal’,	Loss function type, using focal loss
focal_alpha’: 0.5,	Class weighting parameter for focal loss
focal_gamma’: 2.0,	Difficulty adjustment parameter for focal loss
use_ppm’: True,	Enable Pyramid Pooling Module
use_cbam’: True,	Enable Convolutional Block Attention Module
freeze_backbone’: True	Number of epochs to freeze backbone parameters

**Table 4:** Results of ablation experiment (accuracy of test set).

Experiment Group	Accuracy	ΔAcc
Baseline Model	82.5%	-
+CBAM	87.3%	+4.8%
+PPM	85.9%	+3.4%
+Transfer Learning	88.6%	+6.1%
+Focal Loss	89.2%	+6.7%
Full Model (All Components)	92.4%	+9.9%

**Table 5:** Comparison of Classification Accuracy Across Different Networks.

Model	Classification Accuracy	Inference Speed (ms/Sample)
Faster R-CNN + FPN	78%	200 ms
YOLOv4	80%	30 ms
YOLOv5x	82%	25 ms
YOLOv7	85%	35 ms
SSD 513	75%	80 ms
DSSD513	83%	80 ms
DETR	88%	100 ms
ViT-FRCNN	89%	150 ms
ViDT	84%	180 ms
**Our Model**	92.4%	22.3 ms

**Table 6:** Comparison results of multiple models.

Model	Accuracy	Precision	Recall	F1-Score	AUC-ROC	Inference Speed (ms/Sample)
ResNet50	82.5%	81.2%	79.8%	80.5%	0.852	18.7 ms
ResNet50 + CBAM	87.3%	86.1%	85.5%	85.8%	0.891	20.5 ms
ResNet50 + PPM	85.9%	84.7%	83.2%	83.9%	0.878	19.8 ms
Our Model	92.4%	91.6%	90.7%	91.1%	0.945	22.3 ms

**Table 7:** Class-wise Performance of the Optimized Model.

Class	Accuracy	Precision	Recall	F1-Score	Support (Number of Samples)
Good	92.5%	91.7%	93.9%	92.7%	1200
Bad	92.3%	91.5%	90.7%	91.1%	800
Average	92.4%	91.6%	92.3%	91.9%	2000

## Data Availability

The raw data supporting the conclusions of this article will be made available by the authors on request.
